# Fad, Fact and Fancy in Medicine1President’s address before the Thomas J. King Medical Club.

**Published:** 1900-03

**Authors:** Clarence King

**Affiliations:** Machias, N. Y.


### FAD, FACT AND FANCY IN MEDICINE.[1]

By CLARENCE KING, M. D., Machias, N. Y.

FOLLOWING an old and well-established practice which seems to be common to nearly all scientific and many social and religious societies, it becomes my duty at this time to read to you a paper or essay upon some general subject, which you, by courtesy, is called an "address." Just how, or when, or where the custom originated which imposes this duty upon the retiring president I am unable to tell, and it would be difficult to ascertain correctly. But

1. President's address before the Thomas J. King Medical Club.

it affords me an opportunity to express my gratitude to the members for the patience and forbearance they have shown me, and also for the zeal with which they have responded and assisted me in my efforts to establish a new medical society. I sincerely hope that under the guidance of my successor this society will prove itself worthy of and will receive the support of every physician within the territory it seems to cover. But we must remember the Club will be what its members make it; hence, we should all strive to attend every meeting and make every effort to raise its literary and scientific standing to as high a plane as possible.

Our Club was organised one year ago for the purpose of bringing together in closer professional and social relation those physicians residing in the adjacent towns of Erie, Wyoming, Allegany and Cattaraugus counties who have heretofore been separated from each other in their society work by the sharp distinction of county lines. It was not our intent to injure the older societies, but on the contrary, we expected to help them by arousing a new interest in such work among those who heretofore have rarely attended the county societies. The Club thus far has more than met our expectations, but there are still a few who hold aloof from us for reasons best known to themselves. These we should again invite, personally, if possible, to join us, and point out to them the benefits which come from a smaller society which meets often, and at the same time let us assure them, both by word and action, of our loyalty to the older societies.

. . . . . . . . . .

Having said all I care to in regard to our club and its prospects, I will now turn to my address proper. The subject I have chosen may not be of practical benefit to our patients, but I hope to make it of temporary interest to you at least.

If we turn back the pages of sacred history, we find that at one time the Creator of the universe commanded that there should be light and there was light. Then He collected the waters of the earth together and caused the dry land to appear, after which He brought into being every form of vegetation, the fishes of the seas, the birds of the air and the beasts of the land. Finally, the record tells us, He made man in His own image and gave him dominion over the fishes of the sea, the fowls of the air and everything which is upon the earth. From this we see that light, and we infer heat, electricity, gravitation and the other invisible forces of nature, existed for many centuries before the birth of man; yet it is only in comparatively recent times that the importance of these has been recognised and an attempt made to turn them to practical utility. Moveover, while man was given dominion over the earth and everything upon it, these forces or phenomena of nature have ever eluded his grasp, although he is constantly trying to bring them also under his control. Some of these have already been put to some use, but much remains for succeeding generations to accomplish. Research in the field of electrical science is just now being conducted with remarkable vigor, and who can tell but that the electric current may be the lever, resting upon the fulcrum of human ingenuity with which some Archimedes may yet move the earth from its accustomed orbit? So, in developing the electric fad, we may be working upon dangerous lines, for if the equilibrium of the universe should be destroyed, we might be rushed to a doom more terrible than the confusion of tongues or even than the deluge.

A fad may be defined as a novel idea or a peculiar habit or method which suddenly becomes popular with a large number of people, generally at the neglect of other subjects. It differs from a hobby only in extent. Thus, a hobby might be called an *endemic* peculiarity, as it attacks only one individual and often remains permanently; while a fad may be the same peculiarity after it has become *epidemic* and infected a large number of people over a wide extent of territory, but which soon disappears and is followed by a new one. A hobby may always remain a hobby, or it may spread to others and thus become a fad. Likewise, a fad may continue a fad until it gradually dies out and disappears, or it may become established as a fact and prove a valuable addition to scientific knowledge and of inestimable worth to thousands of human beings. The antiseptic treatment of wounds, which originated with Lister, but was quickly adopted by his colleagues and soon became the popular practice among surgeons of the whole civilised world, may be cited as an illustration of a fad which proved more than a worthless theory. And if we will scrutinise history closely we will find that nearly every truth, every discovery and every important invention passes through at least one, if not both of these preliminary stages before it assumed its true place. Some few discoveries, it is true, have been made by chance and have immediately taken their proper place without any popular clamor, but ordinarily this is not the case. A person to achieve success must have an absorbing interest in his work, in other words must make it a hobby; and there are always plenty of people to endorse any new announcement or change of custom, especially if it promises to become a popular or fashionable fad. Every profession, every trade, and every occupation has its own peculiar fads, but the more progressive the calling the more it has. Consequently the profession of medicine is full of them. They are as numerous as the rays of light which sparkle from the dazzling sun and most of them almost as transient. Each one appears suddenly and shines brightly for a time and lo! you wink, and it is gone. But another one quickly takes its place, just as bright and attractive as the one before which in its turn is soon lost sight of, unless perchance further thought and careful study sift it out as a kernel of truth among the chaff of nonsense. And thus, probably, it will be to the end of time or until human nature undergoes a radical change and the spirit of rivalry or the desire to win applause or public favor shall die out and become lost.

Fads may be divided into three general classes: First, the harmless fads which under no circumstances will injure anyone; second, the indifferent fads, which ordinarily are harmless but under certain circumstances may become harmful; and, third, the dangerous fads or those which are prejudicial to life or perfect health. It may seem strange that our profession, whose vocation it is to preserve life and restore health can entertain any fad of a dangerous nature, but a moment's reflection will convince us that it does. We have all at some time been guilty of some of them, probably under the belief that we were doing the best thing possible for our patients by following the latest and at that time most popular treatment; but further experience may have demonstrated that this particular line of treatment had absolutely no merit, or even nullified what chances there were of a speedy restoration to health. It is impossible for us to test clinically every theory and every remedy as they are announced, for they are legion and time and opportunity are wanting. We would do well to make the following the rule and axiom of our practice: Never be the first to adopt a new and radical theory or line of treatment nor yet the last. We better leave to those whose individual cases are securely hidden in a large hospital the experimenting in new and untried fields, while we cling to the truths and methods which have stood the test of careful trial and mature approval. By so doing, it is true, we will not come into sudden notoriety or have our names heralded far and wide as prospective benefactors of our race, but we will escape that severe criticism which always follows when it is believed the doctor is "experimenting" with human life. The country doctor's practice is always open to public scrutiny and his failures and his successes are alike known to all, however much he may try to hide them; and gossip often adds to the one and prejudice detracts from the other, both to the doctor's disadvantage.

A fad may be thought harmless at one time, but later be recognised as dangerous, and it is for this reason that many fads are dropped. Likewise, some fads which might be classed as indifferent or even dangerous at first may become harmless when properly restricted by further study. The operative treatment of appendicitis may be given as an illustration of the latter, for while urged as the proper treatment for every case, it was dangerous as it included many cases which did not require operation; but when restricted to selected cases it has become as nearly harmless as any severe operation can be.

Medical fads of the first class, pure and simple, are few. If asked to name some of them we would all have to reflect awhile and probably no two of us would agree. Perhaps, climatic treatment is as good an illustration as we can give, as it is seldom or never attended with harmful ıesults which can properly be charged to the treatment. The foreign fad, which supplies our journals and text-books with foreign abstracts and quotations, furnishes our laboratories and dispensaries with foreign chemicals and drugs and fills foreign clinics with American students, is another fad of this class. Then there is the fad for conjoint labor, so popular with publishers and exemplified by the various "systems" and "encyclopedias" with which the market is flooded. All these are planned with a thought to their future sale rather than to merit and must necessarily be somewhat broken and inharmonious in their context.

The harmless fad is also often a silly fad. From time immemorial people have been seeking for the fountain of perpetual youth where the ravages of time and the cares of age might be effectually washed away. The reputed nectar of the gods has been sought for far and wide but never found. Oh! what joy greeted the announcement from Paris that at last the precious remedy had been discovered and that to use it would surely be to turn back the dial of our lives. No longer was the aged sire to totter beneath his burden of three score years and ten but by this potent testicular fluid he was to become a youth of twenty. But alas, what a pity! the sands keep running and father time is ever busy with his scythe just the same as before. That silly fad soon died out, but it left one offshoot which promises better things because its claims were not so extravagant.

Next followed the Burgeon fad for the treatment of tuberculosis, harmless at first, but dangerous afterward, because it temporised with time which is valuable to the consumptive at the beginning of his disease. On this account it can be given as an example of the second class of fads, which is by far the largest class. This treatment is sought to neutralise the tubercular infection and supply oxygen by transforming the rectum into a breathing apparatus, but the rectum rebelled and refused to serve two masters. It soon became evident that oxygen was not the specific and that we might as well ask the healthy lungs to digest food as to expect the intestines to breathe; and soon asthetic people bolted on the whole theory as it brought no beneficial results whatever. And so this stands as another lamentable disappointment to the consumptive.

The third class of medical fads, or those which are dangerous to life or detrimental to health, are also very numerous. We can all cite illustrations by the score. Calomel used to be the panacea for every illness and salivation was no novelty. The lancet had its day and many patients were undoubtedly bled into their graves. Now, patent medicines seem to be a popular fad with the people. Their name is legion and their value small, although each may be of some use in certain cases. Then there is the twin fad to the patents which belongs to the profession as the patents belong to the people. I refer to the semi-proprietary remedies that are "sold only on physician's prescriptions," or "advertised only in medical journals." Our office tables are covered with reprints extolling them and giving clinical reports, covering fully as wide a range of diseases as is claimed for the patents. But while it requires fine discriminating powers to see the difference between these and the patients (for both are secret or might as well be), these pass as ethical and receive the sanction of the profession while the patents do not.

A few years ago the fad for oöphorectomy broke out and spread faster than measles or the grippe. Every tyro who had seen a case had to go to cutting and collecting ovaries in a jar; then he published the results of his labors, which showed him an expert and multitudes flocked to him for operation. Science and fashion demanded the sacrifice and the poor woman freely offered her ovaries upon the altar (or table) of the operator in support of her faith and the surgeon; but how many of those ovaries might have been left in their native element no one can tell.

In 1890, Koch, of Berlin, published a notable paper in which he announced the discovery of a new treatment for tuberculosis, the nature of which he for a time kept secret. This paper was followed by a second and later by a third communication, each promising such marvelous results that they set the whole world in a turmoil to get full information and a supply of the wonderful lymph for trial. Medical journals published full reports to the exclusion of nearly everything else and societies called special meetings to send delegates to Berlin to investigate. Others went on their own account in a mad race to learn and get back, so as to distance all rivals and secure a rich clientèle. Thus the elite of the whole profession from the four corners of the globe made their pilgrimage to Berlin, like monks to the Holy City, and cast themselves at the feet of Koch and begged for tuberculin. For the time being Berlin was the most famous city in existence for it contained the balm of Gilead which was sought for by thousands. But, alas! our hopes were to be again shattered by disappointment. At present tuberculin is seldom used, except in lupus and as a means of diagnosis in veterinary practice, but the great number of tuberculin syringes now out of use, bear witness to the former popularity of this fad.

Again, there is antitoxin which has absorbed public interest for some time past, but is now dying out somewhat as a *fad*, because the novelty has worn off. Possibly this should be mentioned as a fad of the second class rather than the third, for certainly it can produce ill-effects if used recklessly. Statistics prove its great value in diphtheria, and under certain restrictions it is destined to occupy a prominent place in the treatment of that disease.

In speaking of the second topic of this address, that is, of fact I shall also consider its opposite—fallacy. There may be some difference of opinion among different persons in regard to fact and fallacy for it is well known we cannot all see things just alike. Lawyers know it is sometimes a difficult task to prove a fact which is plain to everyone. It was a long time before the circulation of the blood became generally recognised as a fact; and the fallacy of regarding insanity as witchcrafty or evidence of the evil spirit was not discerned for many years. One proof of a man's ability in our profession rests upon his capacity to discriminate between fact and fallacy. "Good horse sense" may not mean superior education, but it generally does mean a successful man in whatever calling he may be. Diagnosis demands this natural faculty of good judgment before everything else, for without it book-learning is as naught and treatment founded upon fallacy can not be remedied by the most skilful art of the prescriber. Nature often does more for the patient and the reputation of the doctor than all the pills and powders we can give. This is a fact we must admit, however odious it may sound. Consequently, credit may be given us when none is due, but again we are often censured for bad results where the inexorable laws of fate have been against us, and it was beyond the power of man, whatever his attainments, to change the final results.

We might give numerous illustrations of fallacies in the medical profession, but few will suffice. We should not assume that every case of tuberculosis is necessarily fatal, even if Koch's treatment and Bergeon's method and a hundred others have proved worthless. Tuberculosis of bones and joints frequently result in cure under proper treatment and autopsies show numerous cases of cicatrised cavities in the lungs. Hope ought not to be abandoned with the diagnosis of nephritis, either acute or chronic; nor ought morphia to be administered for every trifling pain or ache. And, finally, it is a fallacy to believe that a physician's time and thought should be devoted wholly to his profession, year after year, without vacation. Relaxation from labor brings recuperation of strength, whether it has been the mind or the body which has been taxed. And a month's respite from his ordinary cares and trials spent in travel or pleasure far from home will bring renewed health and strength and add years to his life of usefulness.

Fancy in medicine is my third topic and I imagine you admonish me to be brief. Fancy is an attribute belonging to poets and novelists, and physicians rarely think they have the opportunity to indulge it. The science of medicine deals with the realistic and not the imaginative and its practice requires hard labor and constant application to duty. However earnestly we may wish success or favorable results they seldom come to us except through our own efforts, and even then they often are denied us. We can not plan our lives, as authors do their novels, and expect the future to bring out all the details in accordance with those designs. But as a pastime we can indulge our fancy in weaving day dreams or speculating on events to come and many a tedious and lonely ride can be made shorter and less tiresome by this diversion. If the masters of old could arise from their graves and look about them now they would imagine they had been transported to fairy-land and that the people around them were magicians, so wonderful are their abilities. Medicine has been a progressive science and has appropriated knowledge from every field to increase its usefulness. Its members have won for themselves great distinction for their attainments, their devotion to their work and their zeal in hunting out new truths and applying them. Already the bright day-star of their fame has arisen and is steadily ascending toward the unclouded zenith, wherever that may be. But who can foretell what progress the next generation will produce? Electricity, which is now the motor-car to convey thought and sound between distant places, may yet be employed to carry rays of light which will enable us to see our scattered patients while still sitting in our office chairs. Some wise legislator may yet perfect a law which shall protect us from our greatest enemies; and the substituting druggist, the meddlesome neighbor and the various humbugs which combine to rob us will trouble us no more. Other wonders in different lines will doubtless be accomplished. Many diseases will be legislated out of existence by the sanitarian and others will lose their terrors. No one can believe the triumphs of surgery are exhausted yet and we can speculate upon its next successes. Already the abdomen and pelvis have been invaded by the knife and nearly every organ removed in part or entire. Perhaps the vital organs within the chest will next receive attention and operations upon the heart and lungs be successfully performed. Other operations will, of course, be devised to replace some now employed, which no doubt will surpass in novelty of conception, boldness of execution and final success any of those with which we are now acquainted.

And now I will indulge one more fancy and I am done. Possibly you consider those I have mentioned as doubtful; if so, I fear you will think this impossible and say we are drifting farther and farther from perfection, instead of toward it. But I think I see around us many signs which should cause us to rejoice. The superstition of former ages has been cleared away and in its place has arisen the bright orb of progress and reform. The present hour offers brighter prospects and is illuminated by nobler achievements than ever before. For us the golden age is dawning when everyone from the infant in its cradle to the hoary-headed sire beside his grave must feel the advantages of living in this enlightened generation. Few drawbacks there are to mar the splendor of the closing year of the XIX. century and already the horizon is opening out broader and brighter than ever. Civilisation is making rapid progress and man is becoming more refined in his tastes, his customs and his habits. Christianity is spreading in every land and to every people and the vices and errors of life are being encompassed in narrower bounds. But our fancy can paint still brighter pictures for our posterity and our anticipation hope for still better days to come. All things are said to work together for good to those who have faith and therefore let us trust that at some time in the near future, within *our* lifetime if possible, there may come the day when human nature will be transformed and when all persons will be honest from choice. And when that time comes, if it ever does, we think the people will appreciate the exacting and self-sacrificing labors of the medical profession and we will receive the same courteous treatment that is accorded to other occupations. Then, too, the people will realise that we must have compensation for our work and that a doctor's credit is limited like other men's and his purse often needs replenishing. And as the great family of dead-beats, from whom we now suffer most, will then be extinct, so then it will not be necessary for us to invoke the saber or the lash or the courts of law to obtain recompense for services faithfully performed. Fortunate, indeed, will they be whose lot is cast in such an age for it would usher in the millennium when all men would be made perfect.

